# Multiple *Brucella melitensis* lineages are driving the human brucellosis epidemic in Shaanxi Province, China: evidence from whole genome sequencing-based analysis

**DOI:** 10.3389/fcimb.2024.1452143

**Published:** 2024-10-30

**Authors:** Cuihong An, Shoumin Nie, Boyan Luo, Dijia Zhou, Wenjing Wang, Yangxin Sun, Suoping Fan, Dongli Liu, Zhenjun Li, Zhiguo Liu, Wenhui Chang

**Affiliations:** ^1^ Department of Plague and Brucellosis, Shaanxi Center for Disease Control and Prevention, Xi’an, China; ^2^ National Key Laboratory of Intelligent Tracking and Forecasting for Infectious Diseases, National Institute for Communicable Disease Control and Prevention, Chinese Center for Disease Control and Prevention, Beijing, China

**Keywords:** brucellosis, *Brucella melitensis*, species/biovars, WGS-SNPs, genomic

## Abstract

**Introduction:**

Human brucellosis is a severe public concern in Shaanxi Province, China, and investigating the epidemiological relationship and transmission pattern of *B. melitensis* is necessary to devise control strategies.

**Methods:**

In this study, a conventional bio-typing approach and whole genome sequencing of single-nucleotide polymorphisms (SNPs) were employed to identify 189 strains.

**Results:**

Based on the conventional bio-typing, 189 *Brucella* strains were identified as *B. melitensis*, of which 14 were in bv. 1, 145 were in bv. 3, and 30 were variant, and the *Brucella* strains were distributed in all ten cities in Shaanxi Province. SNP analysis was used to identify genetic variation in 189 *B. melitensis* genomes, and maximum-likelihood was used to generate a phylogeny that identified two clades (A and B) and 19 sequence types (STs). The two clades were highly diverse and exclusively of Eastern Mediterranean origin. Clade B contained 18 STs (2-19), with most isolates originating from a broad swath, implying that multiple *B. melitensis* lineages circulated in Shaanxi. The 19 STs were composed of 3 to 46 strains isolated from different counties and years, suggesting that multiple cross-county brucellosis outbreak events are driven by multiple *B. melitensis* lineages. Global phylogenetic analysis revealed that clade A was close to GTIIb, and clade B was placed in the GTIIh lineage, expanding the known diversity of *B. melitensis* from China.

**Conclusion:**

The human brucellosis epidemic in Shaanxi is driven by multiple indigenous circulating *B. melitensis* lineages, the knowledge of which will contribute to devising a control strategy and providing the foundation for a comprehensive regional phylogeny of this important zoonotic pathogen.

## Introduction

Brucellosis is a zoonosis worldwide that has a serious effect on domestic productivity and human health (Rubach et al., 2013). *Brucella* spp. causes acute febrile illness and potentially debilitating chronic infection in humans, which has a serious negative effect on human health and quality of life. At present, there are 12 *Brucella* species found globally that cause human brucellosis cases, including six classical species (*B. melitensis*, *B. abortus*, *B. suis*, *B. canis*, *B. ovis*, *B. neotomae*) and six novel *Brucella* species (*B. ceti*, *B. pinnipedialis*, *B. inopinata*, *B. microti*, *B. papionis*, and *B. vulpis*) originating from mammals and other hosts (Occhialini et al., 2022), and there is one recent potential novel species (*B. amazoniensis*) isolated from smugglers of gold prospectors from the Amazon region of Brazil (About et al., 2023). However, three species including *B. melitensis*, *B. abortus*, and *B. suis* are responsible for most human brucellosis (Darbandi et al., 2022). In particular, two biovars (bv. 1 and bv. 3) from *B. melitensis* are the dominant species globally and trigger human brucellosis cases, which frequently occur and re-emerge (Refai, 2002; Zhu et al., 2020). The highest burden of human brucellosis has been observed in Asia and Africa (Wareth et al., 2022). In China, the brucellosis epidemic pattern has changed, with an expansive geographic trend from north to south (Lai et al., 2017). The epidemic situation exhibited a graduate worsening trend when a total of 69,767 cases were reported from 2,083 counties in mainland China in 2021, a 47.7% increase from 2020 (47,425) (Yang et al., 2023). Shaanxi is located in the hinterland of China, adjacent to eight provinces/areas: Shanxi and Henan in the east; Ningxia and Gansu in the west; Sichuan, Chongqing, and Hubei in the south; and Inner Mongolia in the north, the majority of which are historic brucellosis-endemic regions. Animal trade and cross-border movements are not strictly controlled and are frequent between these areas (An et al., 2022).

Currently, there is an ongoing increasing trend in the seroprevalence of human brucellosis from 2008 to 2020 in Shaanxi Province (An et al., 2022), and the number of affected counties increased from 36 in 2008 to 84 in 2020, with geographic expansion gradually toward southern areas (An et al., 2021). Genome epidemiology based on the whole genome sequencing (WGS) of circulating strains is a critical step in understanding the epidemiological relationship and transmission pattern of *B. melitensis* for further implementation of control measures (Xue et al., 2023). Single-nucleotide polymorphisms (SNPs) are considered the gold standard typing scheme for *Brucella* and are used to delineate pathogen transmission routes for disease surveillance (Janowicz et al., 2018). Therefore, the purpose of this study was to use conventional bio-typing assays and WGS-SNPs to illustrate the epidemiological relationship and transmission pattern of *B. melitensis* strains from Shaanxi Province, China, for the better implementation of evidence-based targeted surveillance and control measures.

## Methods

### Source of strains and the isolation, identification, and preparation of genome DNA

A total of 189 *B. meliteneis* strains were isolated and identified from human blood samples (n=188) and a goat sample (n=1) from 2013 to 2022, including two in 2013, seven in 2014, six in 2015, eight in 2017, 13 in 2018, 27 in 2019, 30 in 2020, 57 in 2021, and 39 in 2022 (**Supplementary Table S1**). All of which were isolated from surveillance samples during this period. The strain from the goat sample was involved in an outbreak event (OE2, S2021) and was related to two (S2019 and S2017) of the human cases. Sampling, isolation, and identification were performed according to the standard bacteriological approach (Araj, 2010). Briefly, 5-10 ml of fresh blood was injected into a biphasic culture flask and incubated at 37°C for at least 30 days continuously and the flask was observed and shaken the flask gently every other day. Suspected colonies were further subcultured and identified using conventional bio-typing (Liu et al., 2017) and AMOS-PCR (Bricker and Halling, 1994). Subsequently, single colonies from each culture of these strains were picked and subcultured for 48h and the strains were collected for genome preparation. Then, with reference to a previous study, pure colonies were harvested in 500 µL nuclease-free water and inactivated at 80°C for 30 minutes (Xue et al., 2023). DNA extraction from these strains was performed using the QIAamp DNA Mini Kit (Qiagen, Germany) following the manufacturer’s protocol for gram-negative bacteria. Furthermore, DNA quality was assessed by Qubit (Thermo Fisher Scientific, USA), and it was demonstrated that the resulting product was of good yield and purity.

### Genome sequencing, analysis, and visualization of the SNPs

The genome sequences of 189 strains were obtained as previously described (Li et al., 2020). Briefly, the DNA sample was fragmented by sonication to a size of 350bp, and the DNA fragments were then end-polished, A-tailed, and ligated with the full-length adaptor for Illumina sequencing with further PCR amplification. Basic quality control metrics for the raw sequence data were generated using FastQC (https://github.com/s-andrews/FastQC) and the reads were trimmed using FASTQ (Chen et al., 2018) (https://github.com/OpenGene/fastp) to remove low-quality reads and adapter sequences. The high-quality clean data were aligned to contigs and further complete assembly was conducted using the SOAPdenovo method (Li et al., 2010). For SNP analysis of the 189 *B. melitensis* genomes, as previously reported (Janowicz et al., 2018), SNP quality was calculated using SAMTools (Li et al., 2009), and SNPs were filtered out if the quality was below 30 or if they were within the vicinity of 10 bp of another SNP. The maximum-likelihood trees of the 189 *B. melitensis* strains were calculated using TreeBeST based on the Maximum-Likelihood Phylogenies (PHYML) algorithm with 1,000 bootstrap replicates (Vilella et al., 2009). Furthermore, a global genetic relationship comparison with 264 strains was conducted with the aforementioned algorithm methods, of which there were 75 strains (**Supplementary Table S2**) from a previous study (Pisarenko et al., 2018). The tree was visualized and edited using iTOL (Interactive Tree Of Life) v6.5.7 (Letunic and Bork, 2021).

## Results

### Identification and differentiation of *Brucella* strains in this study

In this study, 189 strains were identified and differentiated by conventional bio-typing (**Table 1**) and further confirmed as *B. menliensis* by the AMOS-PCR assay. In total, 188 strains were isolated from human blood, and only one strain was isolated from a goat. The ages of the patients ranged from 4 to 80 years. The majority of the strains (85.10%, 160/188) were isolated from patients aged 30 to 69 years. Of the samples, 1.6%, 4.2%, 3.2%, and 4.7% were isolated from patients aged < 10, 10–20, 20–30, and > 70 years, respectively. The majority of the patients were male (n=140) and 48 were female. Most patients (94.7%, 178/188) had close contact with sheep/goats, with the most common clinical manifestations being fever (59.72%, 86/144), fatigue (46.53%, 67/144), sweat (40.97%, 59/144), and arthralgia (44.44%, 64/144), and 8.3% (12/144) of the patient were asymptomatic. In this study, 87.77% (165/188) of the strains were isolated from farmers, eight strains were from students, and the remaining strains were obtained from workers, farm technicians, and veterinary personnel.

**Table 1:** Districts and species/biovar distribution of *B. melitensis* strains in Shaanxi Province.

Areas	Districts	bv.1	bv.3	variant	No. of strains	Total
Northern Shaanxi (North)	Yulin	1	26	2	29	52
	Yan’an	–	20	3	23	
Guanzhong (Middle)	Baoji	2	5	4	11	117
	Tongchuan	2	6	1	9	
	Weinan	4	40	5	49	
	Xi’ an	–	4	1	5	
	Xianyang	5	26	11	42	
	Yangling	–	1	–	1	
Southern Shaanxi (South)	Ankang	–	5	–	5	18
	Hanzhong	–	2	–	2	
	Shangluo	–	8	3	11	
Unknown		–	2	–	2	2
Total		14	145	30	189	

“-”: No strain.

### Geographical distribution profile of the 189 *B. melitensis* strains in this study

**Figure 1:**
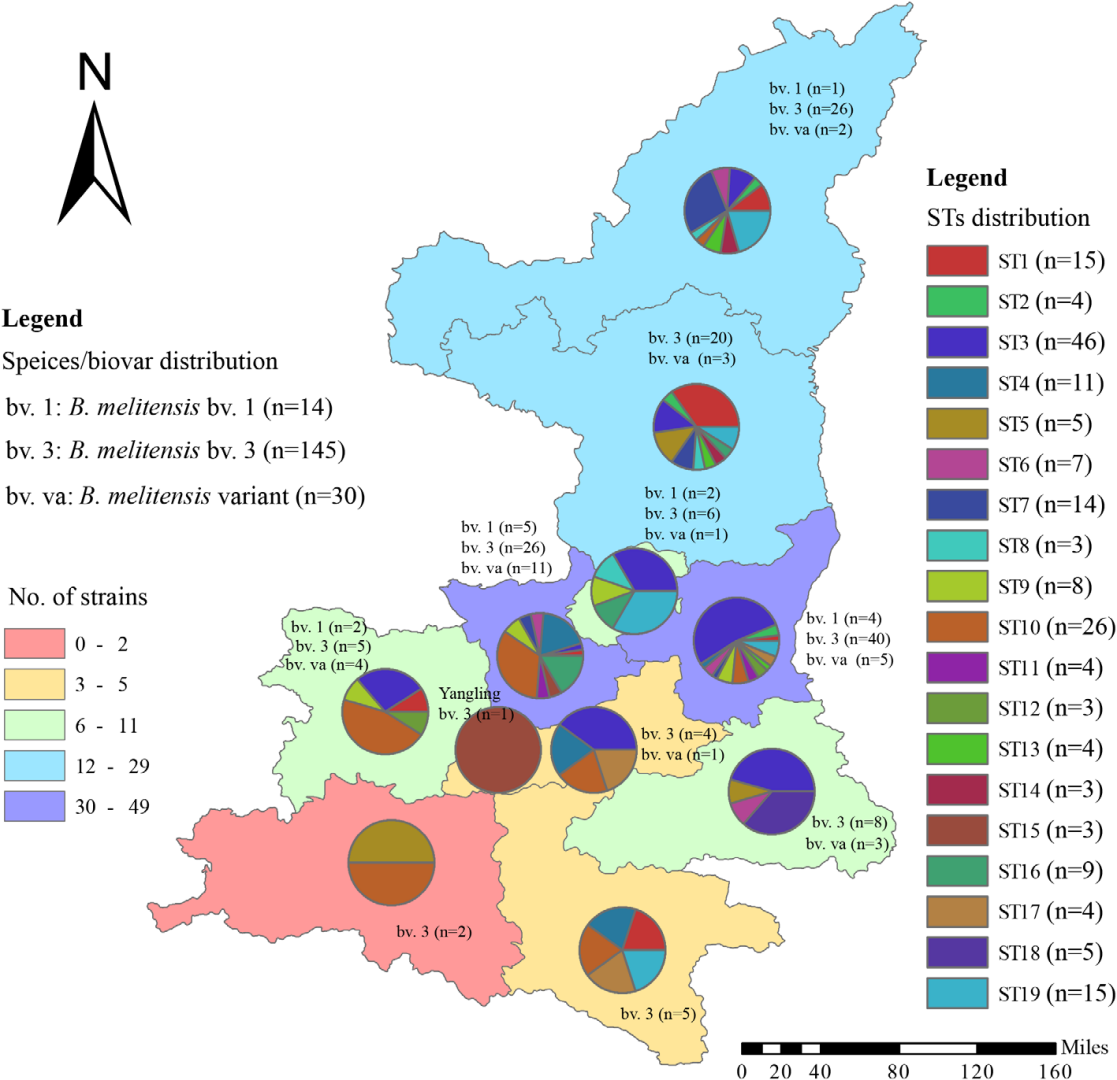
The distribution pattern of the number of strains, species/biovars, and STs of 189 *B*. *melitensis* strains from Shaanxi Province. The red dot indicates that the location of one strain was isolated from a goat.

From 2003 to 2021,189 *Brucella* isolates were obtained from all regions in Shaanxi Province (**Figure 1**, **Table 1**) including 11 cities and 45 counties. Among the isolates, 52 were in Northern Shaanxi (North), 117 in Guanzhong (Middle), and 18 in Southern Shaanxi (South); the location of the remaining two is unknown (**Table 1**). The highest number of strains was found in Weinan (n=49), followed by Xianyang (n=42), Yulin (n=29), and Yan’ an (n=23), and the strain range in other regions was 1 to 11 (**Table 1**). The data show that the source of infection was persistent in this province. Three biovars were found, namely bv. 1 (n=14), bv.3 (n=145), and mutant (n=30), and *B. melitensis* bv. 3 was the dominant circulating biovar (**Table 1**, **Figure 1**).

### Seasonal distribution of *B. melitensis* strains and sequence types

**Figure 2:**
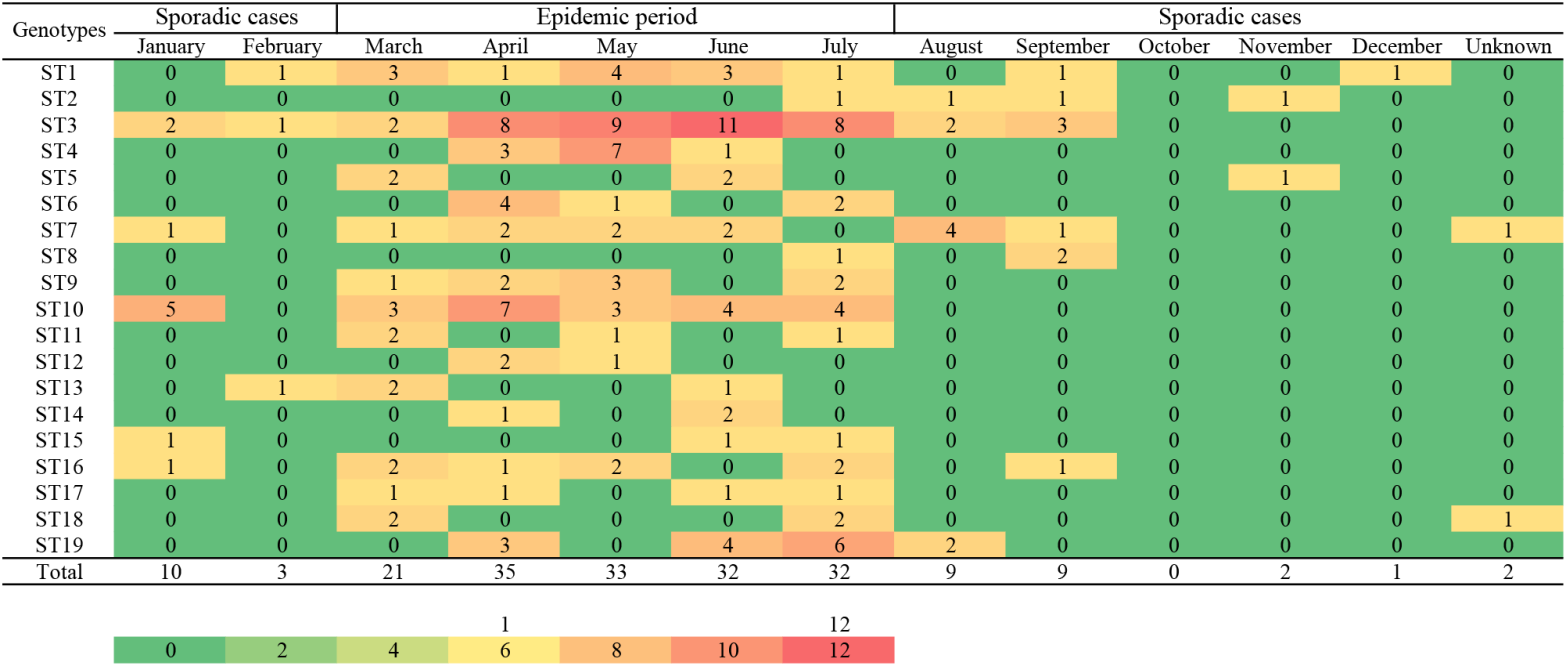
The seasonal distribution profile of the number of strains and STs of the 189 *B*. *melitensis* from Shaanxi Province. A heat map for isolation times of Shaanxi *B. melitensis* strains isolates from 2013 to 2022. The strain isolates were assigned to single nucleotide polymorphism (SNP) sequence types (ST). Color code indicates the number of strains and STs in respective months.

A heat map of the isolation times of *B. melitensis* strains by month showed that most isolates were collected in April (n=35), followed by May (n=33), June, and July (n=32), and the lowest number of strains were collected in December (n=1). Furthermore, 81% (153/189) of the strains were collected from March to July 2013 to 2022 (**Figure 2**). During the same period, the distribution pattern of the sequence types (STs) was similar to that of the strains examined. Some dominant STs had a unique distribution profile; for example, ST1 was dominant in March, May, and June; ST3 from April to July; ST4 in April and May; ST10 in April and January; and ST19 was dominant in July and June. Most STs were distributed during the epidemic period from March to July, with sporadic cases from January to February and from August to December (**Figure 2**). The highest ST diversity of STs was found in Weinan (n=13), followed by Yan’an (n=10), Xianyang (n=10), and Yulin (n=10) (**Figure 1**).

### Genome epidemiology of *B. melitensis* strains based on SNPs

**Figure 3:**
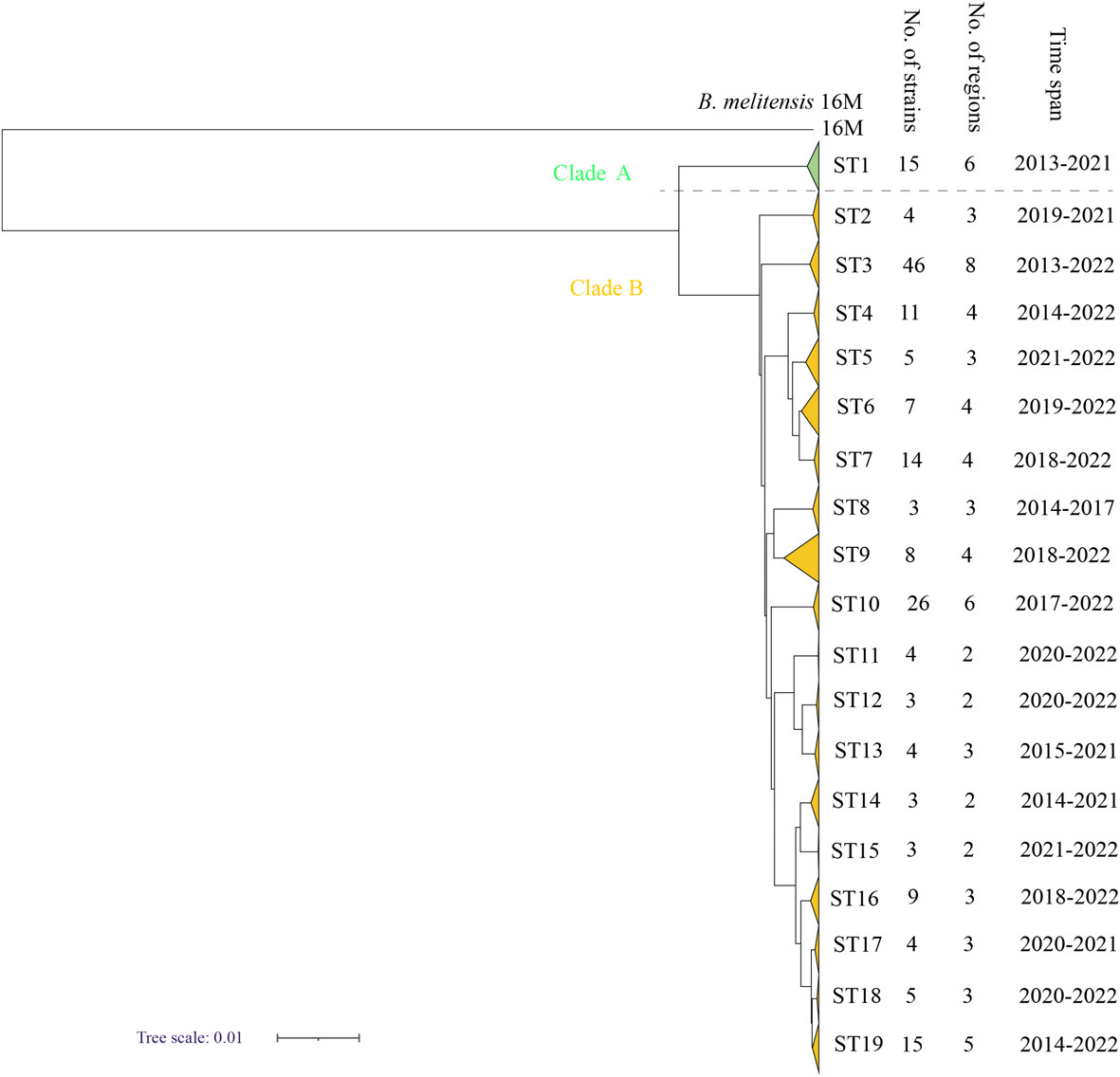
The maximum-likelihood tree generated by the wgSNP matrix of the 189 *B*. *melitensis* on the county scale. The phylogeny trees of 189 *B*. *melitensis* strains were calculated using TreeBeST based on the Maximum-Likelihood Phylogenies (PHYML) algorithm with 1,000 bootstrap replicates.

According to the SNP analysis, the 189 strains from the present study were sorted into two clades (A and B) (**Figure 3**) with 15 strains in clade A and 174 strains in clade B, suggesting that the strains in the present study have two different origins. Furthermore, the 189 strains were divided into 19 STs; ST1 was located in clade A, and the remaining 18 STs belonged to Clade B (**Figure 3**). The strains from each clade consisted of strains from different counties and years (**Figure 1**), and each ST contained between 3 and 46 strains. The most strains were observed in ST3, which contained 46 strains and were collected from eight different counties from 2013 to 2022, followed by ST10, which contained 26 strains from six regions from 2017 to 2022. In addition, there were 15 strains from six regions from 2013 to 2021 in ST1 and 15 strains from five regions from 2014 to 2022 in ST19 (**Figure 3**). These data suggest that multiple cross-county outbreaks of brucellosis were driven by multiple *B. melitensis* lineages. Importantly, most strains from Northern Shaanxi have identical STs compared to strains from Southern Shaanxi, implying that the strains circulating in the south descended from the northern region, demonstrating that *B. meliteneis* strains continuously expanded from Northern Shaanxi towards Southern Shaanxi areas (**Supplementary Figure S1**).

### Trace-back investigation of human brucellosis outbreak events

From 2020 to 2022, 11 human brucellosis outbreak events (OE1-11) were reported in the surveillance system, and a total of 20 strains were isolated and identified (**Supplementary Figure S1**). Among 10 EOs (1-9, and 11), all the strains were grouped in the same STs, which was consistent with the field epidemiology survey, and the strains from each outbreak event were isolated from a common family or patient with close epidemiological links. These data indicated that each outbreak event had a common source of infection (**Supplementary Figure S1**). In contrast, three strains from OE10 were sorted into two different STs; the strains were collected from one family, including the father, wife, and son, and they were all in contact with goats. These data demonstrate that there were two different lineages of *B. melitensis* circulating in this family (**Supplementary Figure S1**).

### Genetic relationship of *B. melitensis* strains on a global scale based on SNP analysis

**Figure 4:**
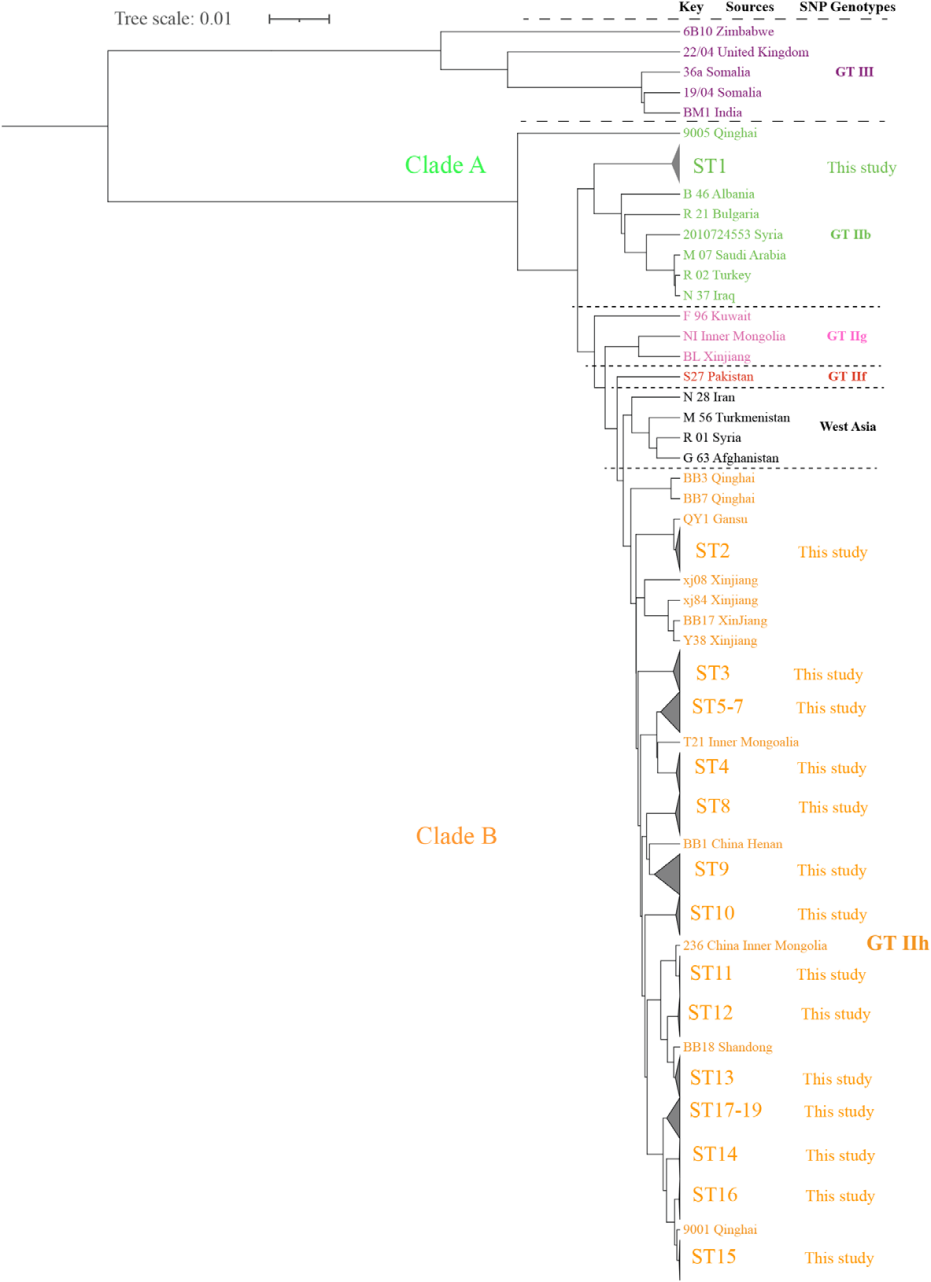
The maximum-likelihood tree of 264 *B*. *melitensis* strains on a global scale. The strains from GT II–III are referred to as previously described (Pisarenko et al., 2018), and GT III are marked with purple, GT IIb with green, GT IIg with pink, GT IIf with red, and the strains from the present study (GT IIh) are marked with orange. The phylogeny trees of the 264 *B*. *melitensis* strains were calculated using TreeBeST based on the Maximum-Likelihood Phylogenies (PHYML) algorithm with 1,000 bootstrap replicates.

Global SNP phylogenetic analysis showed that the 189 strains were placed in genotype II and were still sorted into two clades (A and B) (**Figure 4**, **Supplementary Figure S2**). Clade A contained only ST1, which consisted of 15 strains that were close to strains from GTIIb (2010724553, Syria), and consisted of strains from West Asia and southern Europe, including Syria, Turkey, Iraq, Albania, and Bulgaria, which expands the known diversity of *B. melitensis* in China (**Figure 4**, **Supplementary Figure S2**). Clade B contained 18 STs and was placed in the GTIIh lineage. The strains in these STs had high genetic homogeneity with strains from Gansu, Inner Mongolia, Henan, Shandong, Qinghai, and Xinjiang (**Figure 4**). These are regions with a high brucellosis burden. The ladder-like phylogram of clade B belonging to genotype II suggests a possible single introduction of East Mediterranean origin. The present analysis demonstrated that multiple indigenous imported *B. melitensis* lineages from single ancestors co-drove the continuous human brucellosis epidemic in Shaanxi Province.

## Discussion

In this study, a conventional bio-typing approach and a robust genome epidemiology tool, wgSNP, were applied to identify 189 circulating *B. melitensis* strains from Shaanxi Province, located in the hinterland of mainland China. Our study showed that there have been multiple-point outbreaks of human brucellosis caused by multiple imported and indigenous epidemic *B. melitensis* lineages. This study provides genome-based-evidence showed that animal brucellosis is an epidemic in this period (Chang et al., 2020). Furthermore, the present analysis showed that the epidemic period was concentrated from March to July, which may be because these months represent the time of lambing, shearing, and taking care of the lambs in the agricultural and pastoral areas, leading to frequent contact with animals and increasing the risk of infection (Yu et al., 2023). Human brucellosis occurs throughout the year, with an obvious seasonal increase between March and July (Shi et al., 2017). Thus, appropriate strategies for brucellosis prevention and control should be developed during this high-incidence season. The epidemic of animal and human brucellosis in Shaanxi Province may be caused by a lack of controlled movement and the introduction of infected animals and livestock.

The epidemic trend of human brucellosis in Shaanxi Province is consistent with the majority border northern provinces; the number of cases in 2021 increased by 47.7% compared to that in 2020, and approximately 95.5% of the total disease burden was centralized in the northern provinces, especially Inner Mongolia and neighboring provinces (Yang et al., 2023). Human brucellosis is mainly transmitted by infected livestock, such as in those engaged in professions directly linked to agricultural and livestock activities for goats or sheep (Bernardi et al., 2022). *B. melitensis* is a great challenge for brucellosis control and is distributed worldwide in countries with a high disease burden (Pisarenko et al., 2018). Therefore, enhancing surveillance and controlling the import of small ruminants are important strategies in this region.

In this study, two distinct lineages were identified: clade A was distributed in a limited geographic range, and clade B was widespread throughout all the regions of Shaanxi Province. The lack of geographic expansion of the strains from clade A may be explained by limited host movement (Kamath et al., 2016). To fill this gap in our knowledge, it is necessary to study isolates from animals and conduct a comparative analysis. Interestingly, strains from clade A were similar to strains from GTIIb (2010724553, Syria) (Pisarenko et al., 2018), which consists of strains from West Asia and South Europe, thus expanding the known diversity of *B. melitensis* in China. Whole genome sequencing of Ethiopian *B. abortus* isolates expanded the known diversity of an early branching sub-Saharan African lineage (Edao et al., 2023). Further investigation is needed to provide informative evidence to better understand the genetic diversity of strains in this region. Notably, strains from clade B contained the remaining 18 STs that had high genetic homogeneity with strains from northern areas. The ladder-like phylogram of clade B belonging to genotype II suggests a possible single introduction of Mediterranean origin (Pisarenko et al., 2018). The absence of a clear differentiation according to geographic affiliation between these subgroups confirmed the frequent penetration of *B. melitensis* strains from one province/region to another (Pisarenko et al., 2018). Frequent and active trade and exchange of small ruminants between Shaanxi and other regions of China could promote this process. For example, some areas of Inner Mongolia and Gansu Province are bordered by Shaanxi, which implies that pathogen dispersal in adjacent areas is likely frequent; therefore, further investigation is necessary. The widespread lineage covered a long period and was dominant in Guanzhong, implying that this lineage originated from the development of large-scale goat farming in this area. However, epidemiology surveillance data from animals in this region is lacking. Data from the official veterinary bulletin showed that, in total, 23,399 animal cases were reported in Shaanxi from 2006 to 2021 and the reported number of animal cases per year increased from 248 in 2006 to 4,204 in 2021. In addition, studies reporting isolates from goats and sheep in Shaanxi are rare. One study showed that a total of 110 *B. melitensis* strains obtained from tissue and milk samples from cattle and small ruminants were serologically positive to *Brucella* infection from seven northern provinces (such as Gansu and Shaanxi provinces) and 50 counties (Sun et al., 2017). We have previously demonstrated that 66 isolates of *Brucella* recovered from sheep and yaks in the Inner Mongolia, Xinjiang, Qinghai and Gansu provinces of northwest China in 2015 and 2016, including *B. melitensis* biovar 3 (n = 58), *B. melitensis* biovar 1 (n = 1), *B. abortus* (n = 5), *B. suis* biovar 3 (n = 2), and *B. melitensis* biovar 3, were found to be mainly responsible for sheep brucellosis in northwest China (Cao et al., 2018). These data indicate that *B. melitensis* from small ruminants and cattle is the main driving factor of the human brucellosis epidemics in these regions. Therefore, the increasing trend in the goat population was highly consistent with the incidence of human brucellosis in Shaanxi. The number of goats (in ten thousand) continually increased from 681.32 in 2003 to 723.9 in 2021; however, the number of sheep declined from 195.86 in 2003 to 157.2 in 2021. These data demonstrate that introduced goats are the main reason for the outbreak of human brucellosis, and the transfer and illegal movement of infected goats triggered many outbreaks spanning years with geographic cross-over. Multiple brucellosis outbreak cases among students were caused by consuming raw goat’s milk in this region, which may be the best evidence (An et al., 2021). A meta-analysis showed that the prevalence of brucellosis in ovine and caprine flocks in China increased from 2010 to 2018 (3.2%) compared with 2000-2009 (1.0%), and the pooled prevalence of brucellosis was higher in goat flocks than in sheep flocks in China (Ran et al., 2018). Weak and relaxed surveillance activity in infected animals, the suspension of vaccination or insufficient vaccination of animals, devolvement of the farming economy, livestock products that demand high populations, and fatalities, combined with the inadequate implementation of quarantine and countermeasures for transregional livestock transport and introduction, play a role in driving the widespread expansion (Guan et al., 2018). For example, Xinjiang is one of the largest livestock husbandry sectors in China and has recently experienced an increasing incidence of brucellosis in cattle and small ruminants (Sun et al., 2016), leading to a high incidence of human brucellosis and brucellosis patients who are mainly pastoralists and veterinarians (Zheng et al., 2021). In Qinghai, WGS-SNP analysis demonstrated that an increase in the incidence of human brucellosis may be caused by multiple local circulating lineages (Xue et al., 2023). In Kyrgyzstan, there were different *B. melitensis* lineages, and strains with high similarity to strains from Turkmenistan, Iran, and Turkey (Kydyshov et al., 2022), which is consistent with our findings that human brucellosis outbreaks in Shaanxi are driven by multiple lineages from single introduced ancestors of *B. melitensis* strains. In addition, Iranian *B. melitensis* strains were closely related to those recovered from sheep and humans in Iraq, Afghanistan, Syria, Turkmenistan, and Pakistan. These findings reveal the frequent and uncontrolled livestock exchange that have been introduced between different areas from the past until today. In Egypt, 136 Egyptian *B. melitensis* strains isolated from animals and humans revealed 99 different cgSNP genotypes, indicating several different incidents and sources of infections, probably from animals imported from other countries to Egypt (Holzer et al., 2022). Brucellosis is an emerging and re-emerging neglected regional communicable disease in some regions of Asia driven by movement and trade (Liu et al., 2020). Therefore, there needs to be effective collaboration locally and nationally, and both animal and human health services authorities need to improve their surveillance capacity of the disease and implement strict measures for animal transboundary movement and create one health approach to curb the spread of the disease.

## Conclusion

Our study identified *B. melitensis* as a causative agent of human brucellosis in Shaanxi Province, and multiple different lineages of *B. melitensis* were found to drive the epidemic of human brucellosis in this area. The present conclusion provides vital clues for understanding the geographic distribution and transmission pattern of the strains. As this study focused on isolates of human origin, the identity of *Brucella* species and lineages circulating among animals remains elusive. Implementing animal causative agent surveillance programs and discrimination based on WGS-SNPs are needed to better understand the transmission pattern to facilitate the implementation of targeted control measures to combat brucellosis in Shaanxi and border areas.

## Data Availability

Genome sequences were deposited in the Genome Warehouse in National Genomics Data Center, Beijing Institute of Genomics, Chinese Academy of Sciences / China National Center for Bioinformation, under accession number that is publicly accessible at https://ngdc.cncb.ac.cn/gwh and accession numbers: GWHFGHQ00000000.1, GWHFGMR00000000.1, GWHFGMS00000000.1, GWHFGMT00000000.1, GWHFGMU00000000.1, GWHFGMV00000000.1, GWHFGMW00000000.1, GWHFGMX00000000.1, GWHFGMY00000000.1, GWHFGMZ00000000.1, GWHFGNA00000000.1, GWHFGNB00000000.1, GWHFGNC00000000.1, GWHFGND00000000.1, GWHFGNE00000000.1, GWHFGNF00000000.1, GWHFGNG00000000.1, GWHFGNH00000000.1, GWHFGNI00000000.1.

## Supplementary material

Supplementary Figure 1 Based on the wgSNP matrix, we generated a maximum-likelihood tree phylogeny tree of the 189 *B. melitensis* strains on the county scale. Nine outbreak events are marked with red, EO10 is marked with purple, and EO5 and 11 are marked with green.

Supplementary Figure 2 Phylogenetic analysis of 264 strains on the global scale. Strains from clade **(A)** are marked with green and strains from Clade **(B)** are marked with red.
