# Supplementary material for: Multiple Brucella melitensis lineages are driving the human brucellosis epidemic in Shaanxi Province, China: evidence from whole genome sequencing-based analysis
Source: Front Cell Infect Microbiol. 2024 Oct 30;14:1452143. doi: 10.3389/fcimb.2024.1452143 (PMC11557520; doi:10.3389/fcimb.2024.1452143)

Figure S1 Trace-back investigation of eleven human brucellosis outbreak events (OE1-11).
Note: nine outbreak events marked with red, EO10 marked with purple, and EO5 and 11 marked with green.

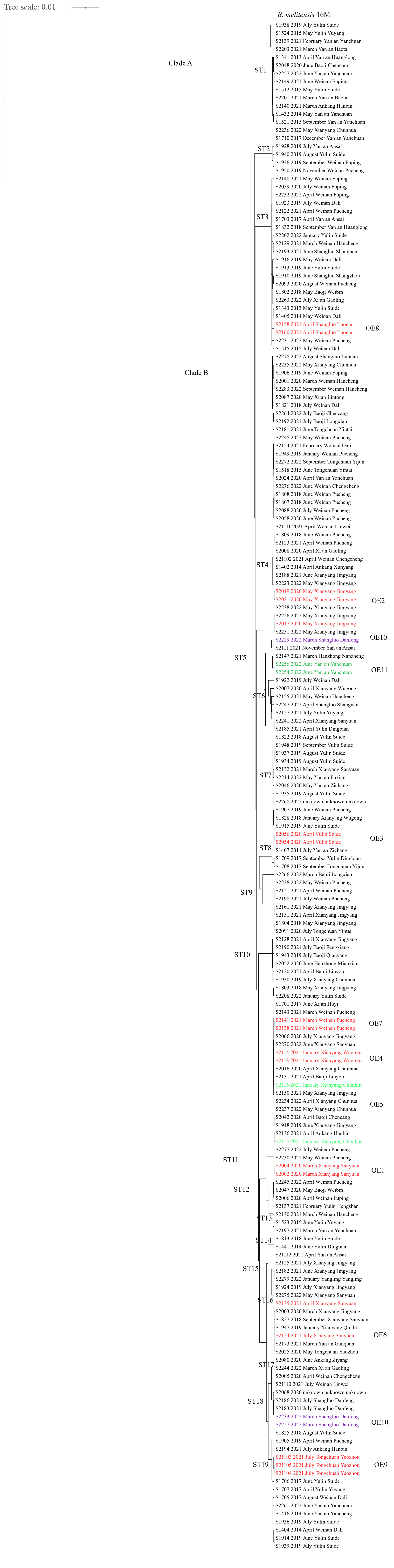

Supplement: Supplementary Figure 1 — Based on the wgSNP matrix, we generated a maximum-likelihood tree phylogeny tree of the 189 B. melitensis strains on the county scale. Nine outbreak events are marked with red, EO10 is marked with purple, and EO5 and 11 are marked with green. [file DataSheet1.pdf]
