# Supplementary figures and images for: Multiple Brucella melitensis lineages are driving the human brucellosis epidemic in Shaanxi Province, China: evidence from whole genome sequencing-based analysis
Source: Front Cell Infect Microbiol. 2024 Oct 30;14:1452143. doi: 10.3389/fcimb.2024.1452143 (PMC11557520; doi:10.3389/fcimb.2024.1452143)

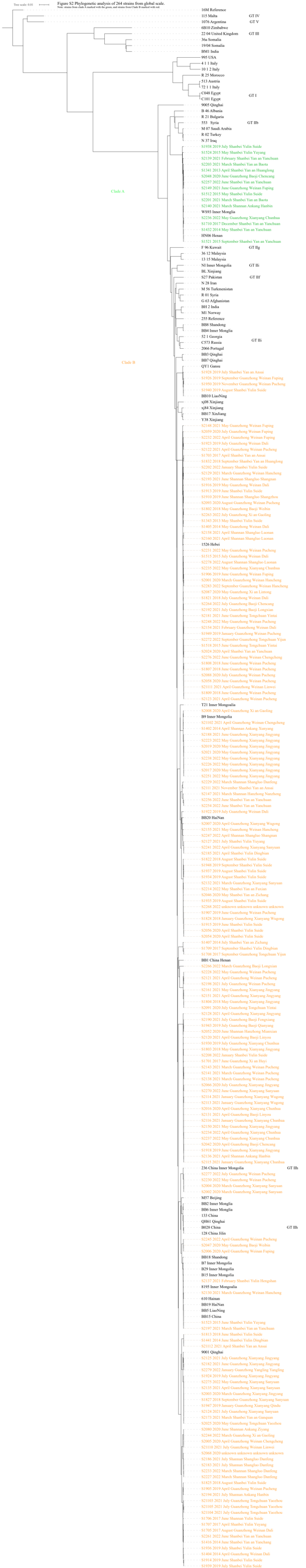

Supplement: Supplementary Figure 2 — Phylogenetic analysis of 264 strains on the global scale. Strains from clade (A) are marked with green and strains from Clade (B) are marked with red. [file DataSheet2.pdf]
